# Mesenchymal stem cells ameliorate experimental arthritis via expression of interleukin-1 receptor antagonist

**DOI:** 10.1371/journal.pone.0193086

**Published:** 2018-02-26

**Authors:** Kijun Lee, Narae Park, Hyerin Jung, Yeri Alice Rim, Yoojun Nam, Jennifer Lee, Sung-Hwan Park, Ji Hyeon Ju

**Affiliations:** 1 Catholic iPSC Research Center, College of Medicine, The Catholic University of Korea, Seoul, Republic of Korea; 2 Division of Rheumatology, Department of Internal Medicine, Seoul St. Mary's Hospital, College of Medicine, The Catholic University of Korea, Seoul, Republic of Korea; University of South Florida St Petersburg, UNITED STATES

## Abstract

Human bone marrow-derived mesenchymal stem cells (MSCs) have been observed to inhibit arthritis in experimental animal models such as collagen-induced arthritis. However, the exact anti-inflammatory mechanisms remain poorly understood. Interleukin-1 receptor antagonist (IL-1Ra) is an anti-inflammatory cytokine produced by immune and stromal cells. We postulated that MSCs could produce IL-1Ra and attenuate experimental arthritis. In this study, 5x10^6^ MSCs were injected into the peritoneal cavity of IL-1Ra knockout (IL-1RaKO) mice. MSCs reduced the severity of the arthritis by histology and decreased pro-inflammatory cytokine levels in IL-1RaKO mice. The ratio of splenic T helper 17 (Th17) cells to regulatory T cells (Treg) was significantly decreased in MSC-injected IL-1RaKO mice. Purified splenic CD4+ T cells from mice in each of the treatment groups were cultured under Th17 polarizing conditions and analyzed by flow cytometry. Less expansion of the Th17 population was observed in the MSC-treated group. Interestingly, MSCs expressed inducible IL-1Ra against inflammatory environmental stimuli. Human recombinant IL-1Ra could suppress Th17 cells differentiation under Th17 polarizing conditions. These results indicate that IL-1Ra expressed by MSCs can inhibit Th17 polarization and decrease the immune response in IL-1RaKO mice. Therefore, MSC-derived IL-1Ra may inhibit inflammation in IL-1RaKO mice via effects on Th17 differentiation.

## Introduction

Rheumatoid arthritis (RA) is a systemic autoimmune disorder characterized by persistent inflammation of the joints and consequent joint dysfunction [1,2]. Although the pathophysiology of RA has not yet been clearly elucidated, many factors contribute to the risk of RA, including genetic background, viral and bacterial infections, and smoking [3–5].

Interleukin-1 (IL-1) is an important factor in the development of RA [6]. IL-1 is secreted by a variety of cells, including macrophages, monocytes, and synovial cells. IL-1 induces various chemokines, cytokines, and inflammatory mediators [7,8]. The IL-1 signal is transmitted intracellularly via IL-1 receptor type 1 [9]. Many studies have shown that IL-1 inhibition alleviates RA [10–14]. These studies imply that IL-1 plays a role in RA pathogenesis. The IL-1 receptor antagonist (IL-1Ra) is a natural endogenous IL-1 inhibitor that blocks IL-1-mediated signaling [15]. Many studies have reported that an imbalance between IL-1 and IL-1Ra is critical in RA [16–18].

IL-1Ra knockout (KO) mice (IL-1RaKO) are an experimental arthritis model designed for the study of RA. IL-1RaKO mice cannot produce IL-1Ra because the IL-1Ra gene is deleted. IL-1RaKO mice are useful for investigating the role of IL-1Ra in the pathogenesis of RA. IL-1Ra knockout BALB/c mice develop spontaneous arthritis that starts at 5 weeks of age and all of the mice became arthritic by 13 weeks of age. [19].

Bone marrow-derived mesenchymal stem cells (MSCs) can differentiate into several types of cells and have the potential for self-renewal [20]. It is also known that the factors secreted by MSCs can suppress immune and inflammatory responses [21]. In clinical trials, MSCs could be promising in the treatment of various autoimmune diseases [22]. Immunomodulation by MSCs is regulated by the secretion of various immunoregulatory factors, including IL-4, IL-10, prostaglandin E2, indolamine 2,3-dioxygenase, and transforming growth factor-beta (TGF-β) [23–25]. It has been shown that IL-1Ra can be expressed by MSCs [26,27]. Recently, MSC-derived IL-1Ra was shown to have an anti-inflammatory effect on B cells and macrophages [28]. In this study, we investigated the role of MSC-derived IL-1Ra on inflammation and Th17 differentiation in the IL-1RaKO model of RA [29]. Intraperitoneal injection of MSCs into IL-1RaKO mice reduced arthritic inflammation by histology and significantly decreased Th17 cell differentiation. This study may elucidate the role of IL-1Ra in the immunomodulatory function of MSCs.

## Materials and methods

### Mouse model

All procedures involving animals were performed in accordance with the Laboratory Animals Welfare Act, the Guide for the Care and Use of Laboratory Animals, and the Guidelines and Policies for Rodent Experimentation provided by the Institutional Animal Care and Use Committee of the School of Medicine of The Catholic University of Korea. The study protocol was approved by the Institutional Review Board of The Catholic University of Korea (CUMC-2016-0097-01). Wild-type (WT) BALB/c mice and interleukin-1 receptor antagonist-deficient mice (IL-1RaKO) on the BALB/c background were kept under specific pathogen-free conditions and fed regular mouse chow and water *ad libitum*. Animals were housed in groups of five and maintained in a temperature-controlled environment at 22°C on a 12:12-h light:dark cycle. Seven-week-old IL-1RaKO mice were intraperitoneally injected with 5×10^6^ MSCs at day 0, followed by an additional injection of 5×10^6^ MSCs at day 43, and then sacrificed at day 70. All mice were placed under gas anesthesia using isoflurane (2–2.5%) and fully anesthetized and sacrificed by cervical dislocation in order to reduce unnecessary pain. All efforts were made to minimize animal suffering.

### Clinical evaluation

The severity of arthritis was scored on a scale of 0–4. Each joint was examined three times a week for swelling and redness. The arthritic score was calculated for the two hind limbs only, giving a maximal score of 8 points for one mouse. For each limb, grade 0 = normal, grade 1 = light swelling of the joint and/or redness of the foot pad, grade 2 = obvious swelling of the joint, grade 3 = severe swelling of the joint, and grade 4 = very severe swelling and fixation of the joint. The extent of arthritis was scored by two independent examiners.

### Mesenchymal stem cells

MSC line (BM025SS13) was obtained from the Catholic Institute of Cell Therapy, South Korea. MSCs were cultured in Dulbecco’s Modified Eagle’s Medium (Gibco/ThermoFisher Scientific, MA, USA) supplemented with 10% fetal bovine serum (FBS) (Gibco) and 1% penicillin/streptomycin (Gibco), and maintained at 37°C in 5% CO_2_. The culture medium was changed twice a week.

### Histological analysis

Mouse hind limbs were removed and fixed in 4% paraformaldehyde (PFA), decalcified in a 10% (w/v) EDTA solution, and embedded in paraffin, and 4 μm-thick sections were cut. For histochemical staining, the sections were stained with hematoxylin-eosin, safranin O, or toluidine blue. Inflammation and joint destruction scores were calculated according to the procedure of *Huckel et al*. The inflammation score was based on immune infiltration and pannus formation, while the joint destruction score was based on cartilage and bone erosion.

### Flow cytometry analysis of Th17 and Treg cells

Mice were sacrificed and their spleens were harvested. The spleen samples were dissociated into single-cell suspensions and 2×10^6^ cells were transferred to round-bottom polystyrene tubes (BD Biosciences, CA, USA). After washing with PBS supplemented with 2% FBS, mouse spleen cells were stained with a rat anti-mouse CD4 (2 μg/mL) antibody conjugated with allophycocyanin (APC) (eBioscience, CA, USA) and a rat anti-mouse CD25 (2 μg/mL) antibody conjugated with APC-Cy7 (BD Pharmingen, CA, USA). The cells were then permeabilized using Flow Cytometry Fixation and Permeabilization buffer (eBioscience), and stained with an anti-human/mouse RORγt (2 μg/mL) antibody conjugated with phycoerythrin (PE) (eBioscience) and an anti-mouse FoxP3 (5 μg/mL) antibody conjugated with fluorescein isothiocyanate (FITC) (eBioscience). Flow cytometry analysis was performed using a BD LSR Fortessa cell analyzer (BD Biosciences). To analyze the data, FlowJo V10 Single Cell Analysis Software was used (TreeStar Inc., OR, USA).

### Reverse transcription-polymerase chain reaction (RT-PCR)

Total RNA was extracted using Trizol reagent (Invitrogen/ThermoFisher Scientific, MA, USA). Then, cDNA was synthesized from 2 μg total RNA using the RevertAid First Strand cDNA Synthesis kit (ThermoFisher Scientific). The following primers were used for RT-PCR: mouse IL-1Ra (forward: 5'-GTGGCCTCGGGATGGAAATC-3', reverse: 5'-CAGCAATGAGCTGGTTGTTTCT-3'); mouse IL-1β (forward: 5'-ATGCCACCTTTTGACAGTGATG-3', reverse: 5'-AGCTTCTCCACAGCCACAAT-3'); mouse IL-6 (forward: 5'-CACGGCCTTCCCTACTTCAC-3', reverse: 5'-CTGCAAGTGCATCATCGTTGT-3'); mouse TNF-α (forward: 5'-CCTCACACTCACAAACCACCA-3', reverse: 5'-GTGAGGAGCACGTAGTCGG-3'); mouse IL-17 (forward: 5'-GGTCAACCTCAAAGTCTTTAACTC-3', reverse: 5'-TTAAAAATGCAAGTAAGTTTGCTG-3'); and mouse β-actin (forward: 5'-AGAGGGAAATCGTGCGTGAC-3', reverse: 5'-CAATAGTGATGACCTGGCCGT3'); human IL-1Ra (forward: 5'-AGAAGACCTCCTGTCCTATG-3', reverse: 5'-TACTCGTCCTCCTGGAAGTA-3'); human β-actin (forward: 5'-AGAAAATCTGGCACCACACC-3', reverse: 5'-AGAGGCGTACAGGGATAGCA-3'). All primers were synthesized by Bioneer Corp. (South Korea). The mRNA levels of various target genes were normalized to the levels of β-actin mRNA.

### In vitro Th17 differentiation

Mouse spleens were harvested and dissociated into single-cell suspensions. The CD4+ T cells were selected positively using anti-mouse CD4 microbeads (Miltenyi Biotec Inc., Bergisch Gladbach, Germany). The sorted CD4+ T cells were cultured in RPMI 1640 medium supplemented with 10% FBS (Gibco) and stimulated with 1 μg/ml plate-bound anti-mouse CD3 (BD Biosciences), 2 μg/ml anti-mouse CD28 (BD Biosciences), 2 μg/ml anti-mouse IL-4 (R&D Systems, MN, USA), 2 μg/ml anti-mouse interferon-γ (IFN-γ) (R&D Systems), 20 ng/ml recombinant IL-6 (R&D Systems), and 2 ng/ml recombinant transforming growth factor-β1 (TGF-β1) (R&D Systems) for 3 days.

### Immunofluorescence and confocal microscopy

The slides were fixed in pre-cooled acetone for 10 minutes and air-dried. Fixed cells were incubated in 0.3% H_2_O_2_ at room temperature (RT) for 10 minutes. The sections were incubated with an avidin/biotin blocking cocktail and M.O.M. mouse Ig blocking reagent (Vector Laboratories, CA, USA) according to the manufacturer’s protocols. The slides were then incubated overnight at 4°C with a 1:100 dilution of a mouse anti-human mitochondria monoclonal antibody (Millipore, MA, USA). They were then washed and incubated with a biotinylated anti-mouse secondary antibody (Vector Laboratories) for 2 hours at RT. For mouse CD4 staining, the sections were incubated overnight at 4°C with a 1:500 dilution of primary rabbit anti-mouse CD4 antibody (Abcam, Cambridge, UK) and stained with an anti-rabbit Alexa Fluor594-conjugated secondary antibody (1:200 dilution). The slides were mounted using ProLong Gold Antifade mounting medium (Invitrogen/ThermoFisher Scientific). Cells were examined under a Zeiss LSM 700 laser scanning fluorescence confocal microscope.

### Immunostaining of MSCs

MSC staining was performed directly in 6-well tissue culture plates. The samples were fixed for 30 min in 4% PFA. The reaction was quenched with 50 mM NH_4_Cl for 10 min. The cells were incubated for 20 min in 0.1% Triton X-100 for permeabilization and incubated for 30 min in PBS supplemented with 2% FBS (Gibco) for blocking at RT. The cells were incubated for 2 hours at RT with a 1:500 dilution of an anti-human IL-1Ra antibody (Abcam), and then for 1 hour at RT with a 1:200 dilution of an Alexa Fluor594-conjugated goat anti-rabbit IgG (H+L) secondary antibody (Molecular Probes, OR, USA). The samples were counterstained with 4', 6-diamidino-2-phenylindole (DAPI) at RT in the dark and examined under a Zeiss LSM 700 microscope.

### Co-culture of Th17 polarized cells and MSCs

MSCs were plated into 48-well plates containing CD4+ T cells differentiated under Th17 polarizing conditions. MSCs were co-cultured at 5×10^4^ cells/ml with 5×10^5^ cells CD4+ T cells (an MSC:CD4+ T cell ratio of 1:10). Human recombinant IL-1Ra (2, 5, and 10 ng/ml) or human recombinant IL-1 receptor type 1 (IL-1R1) was added (2, 5, and 10 ng/ml) under Th17-inducing conditions (see above). After 3 days of co-culture, the T cells were harvested and the proportion of Th17 cells was analyzed by flow cytometry as described above.

### Statistical analysis

All statistical data are expressed as the means ± SEM. The data were analyzed by independent t-tests. A P-value <0.05 was considered significant. In all figures, * indicates P<0.05, ** indicates P<0.01, and *** indicates P<0.001.

## Results

### MSCs ameliorate inflammation in IL-1RaKO mice

**Fig 1:**
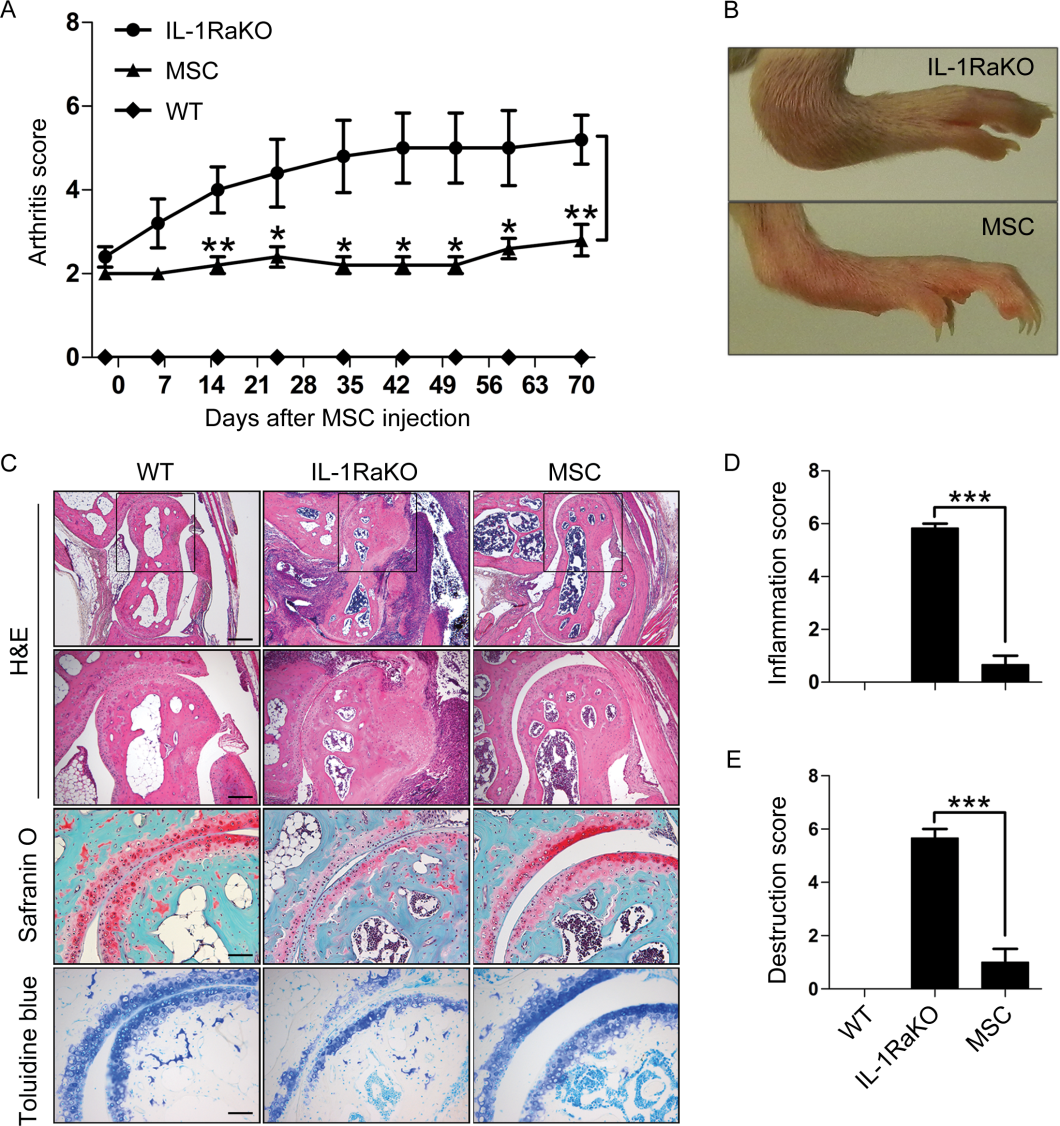
The therapeutic effects of human bone marrow-derived mesenchymal stem cells (MSCs) in IL-1RaKO mice. IL-1RaKO mice were intraperitoneally injected with 5×10^6^ MSCs at 7 and 14 weeks of age. (A) Hind paw arthritis score. Arthritic paws were scored on a scale of 0–4. The arthritis score decreased in the IL-1RaKO mice that received MSCs. (B) Representative photographs of redness and swelling of the hind paws in IL-1RaKO with or without MSCs. (C) Histological analysis of the ankle joints stained with hematoxylin and eosin (H&E), safranin O (S), and toluidine blue (T) in wild-type (WT) BALB/c mice, IL-1RaKO mice, and IL-1RaKO mice injected with MSCs. H&E-stained sections were examined under 50× and 100× magnification. Safranin O and toluidine blue staining was analyzed at 200× magnification. Scale bar: 200 μm (50×), 100 μm (100×), and 50 μm (200×). (D) Synovial hyperplasia and cellular infiltrate were each scored on a scale of 0–3. Joint inflammation scores were calculated as the sum of these two scores. (E) Cartilage loss estimated in samples stained with safranin O and toluidine blue, and the extent of pannus formation, were each scored on a scale of 0–3. Joint destruction scores were calculated as the sum of these two scores. The mean scores were evaluated by three independent examiners. Results are the mean ± SEM. *, P<0.05; **, P<0.01; ***, P<0.001.

To characterize the anti-inflammatory effect of MSCs in IL-1RaKO mice, mice were divided into three experimental groups: wild-type (WT) BALB/c mice, IL-1RaKO mice, and IL-1RaKO mice injected with MSCs (MSC group) (n = 5 mice per group). In the MSC group, mice received intraperitoneal (IP) injections of 5×10^6^ MSCs at day 0 and day 43. Arthritis scores for each mouse were recorded 2 days prior to the first MSC injection. Arthritis was scored on a five-point scale. Compared with IL-1RaKO mice, the arthritis scores of the MSC group were statistically lower at 70 days after MSC injection (5.2±0.6 vs 2.8±0.3, p = 0.004) (Fig 1A). Representative images of hind paws are shown in Fig 1B. A significant decrease in arthritis score was observed in the MSC group. Histopathologic analysis was performed by hematoxylin-eosin (H&E), toluidine blue (TB), and safranin O (S) staining (Fig 1C). H&E staining showed diffuse cell infiltration and pannus invasion in tarsal bone. IL-1RaKO control mice exhibited tremendous damage compared to WT mice, however, joints of the MSC injection group were less invasive. Cartilage damage was confirmed by TB and S staining. The level of cartilage damage was lower in the MSC group than in the IL-1RaKO control group. In addition, the joints of the MSC group had less cell infiltration than the IL-1RaKO control group. Based on these analyses, inflammation and destruction scores were calculated (Fig 1D and 1E). The joint destruction and inflammation scores were both significantly lower in the MSC group than IL-1RaKO group. In conclusion, we confirmed that injection of MSCs relieved the arthritic symptoms of IL-1RaKO mice.

### MSCs decrease the splenic Th17/Treg ratio and levels of pro-inflammatory cytokines in the ankle joint

**Fig 2:**
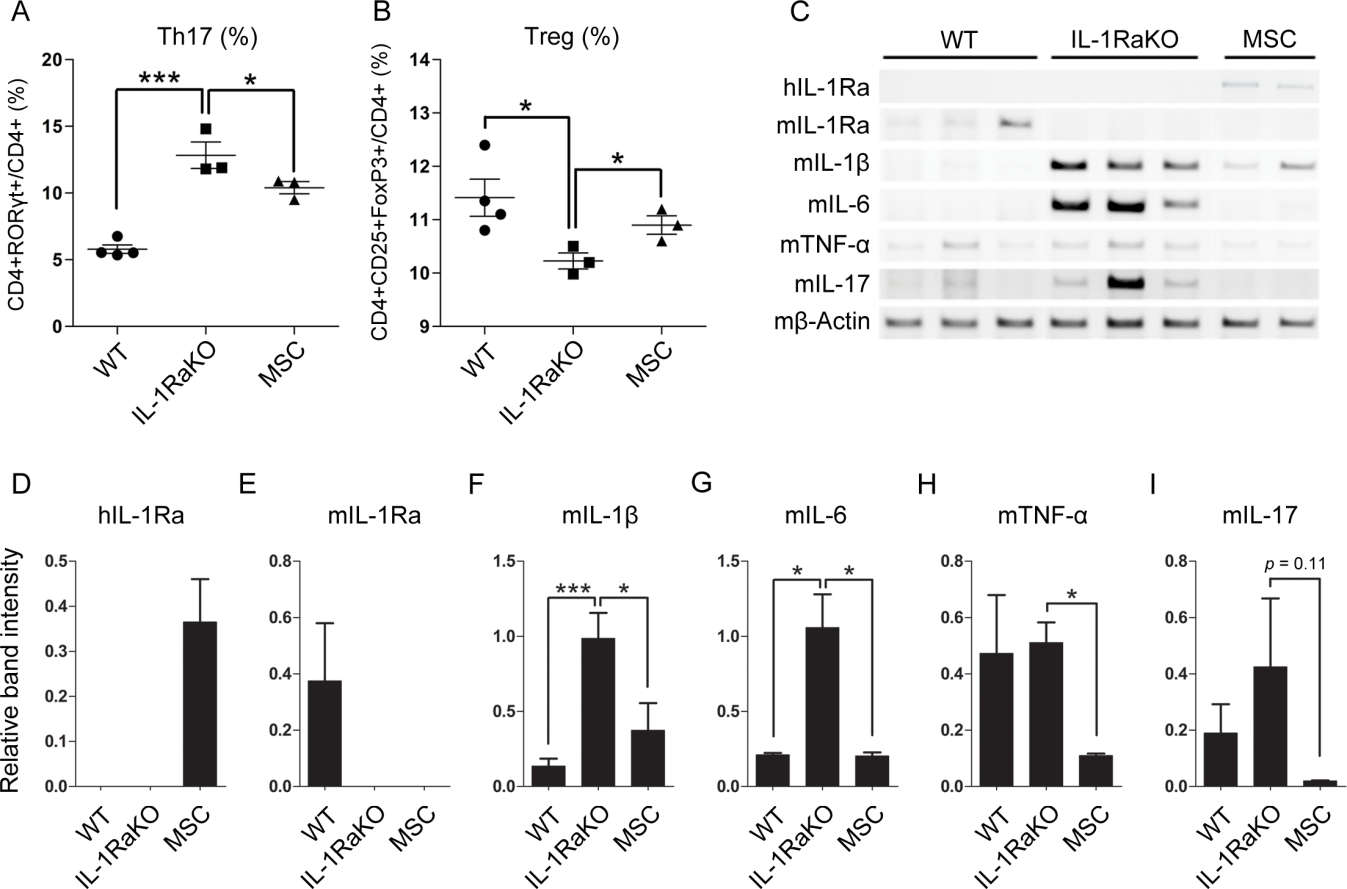
MSCs decrease the splenic Th17/Treg ratio and mRNA levels of pro-inflammatory cytokines in the ankle joint. (A, B) CD4, CD25, FoxP3, and RORγt expression in the spleen were determined by flow cytometry. The frequency of Th17 cells was reported as the percentage of CD4+ RORγt+ cells out of the total CD4+ T cell population. The frequency of Treg was reported as the percentage of CD4+CD25+FoxP3+ cells out of the total CD4+ T cell population. (C) Total RNA was isolated from the joints, and the expression of human, mouse IL-1Ra and pro-inflammatory cytokines such as IL-1β, IL-6, TNF-α, and IL-17 was investigated. (D-I) The densitometric quantification of the mRNA expression in joints. β-actin expression was used as the endogenous control. Data represent the mean ± SEM. *, P<0.05; **, P<0.01; ***, P<0.001.

To determine whether the injected MSCs affected the T cell response in IL-1RaKO mice, the levels of splenic T helper 17 cells (Th17) and regulatory T cells (Treg) were compared by flow cytometry. Th17 cells were defined as CD4+ and RORγt+ T cells, while Treg cells were defined as CD4+, CD25+, and FoxP3+. Th17 was increased in both IL-1RaKO and MSC group. However, the percentage of Th17 was lower in MSC group than IL-1RaKO group (Fig 2A). In the case of Treg population, the decreased population was recovered by MSC injection (Fig 2B). These results are consistent with the previously observed decrease in inflammation. Additionally, RT-PCR was performed to determine the levels of mouse pro-inflammatory cytokines, IL-1Ra, and human IL-1Ra in the ankle joint tissues of IL-1RaKO mice (Fig 2C). The mRNA levels of the cytokines were quantified by densitometric analysis of RT-PCR results. Previous study has shown that up to 30 days of in vivo MSC detection is possible [30]. These results indicate that MSCs migrate to the mouse spleen through the expression of human IL-1Ra even after approximately 30 days (Fig 2D). Expression of mouse IL-1Ra gene was observed in WT but not in IL-1RaKO and MSC group as expected (Fig 2E). The expression of mouse pro-inflammatory cytokines such as IL-1β, IL-6, TNF-α and IL-17 was decreased in the MSC group compared with IL-1RaKO (Fig 2F–2I). IL-1β and IL-6 were significantly increased in IL-1RaKO compared with WT, and it was confirmed that MSC treatment reduced IL-1β and IL-6. In the case of TNF-α, there was no significant difference between WT and IL-1RaKO, but it was significantly decreased when MSCs were injected. IL-17 was increased in IL-1RaKO compared with WT, and decreased when injected with MSCs, but there was no significant difference (p = 0.11). These results confirmed that MSCs migrated to spleen in mouse in vivo, and inflammatory environment was changed.

### MSCs interact with mouse CD4 cells and down-regulate Th17 cell differentiation in IL-1RaKO mice

**Fig 3:**
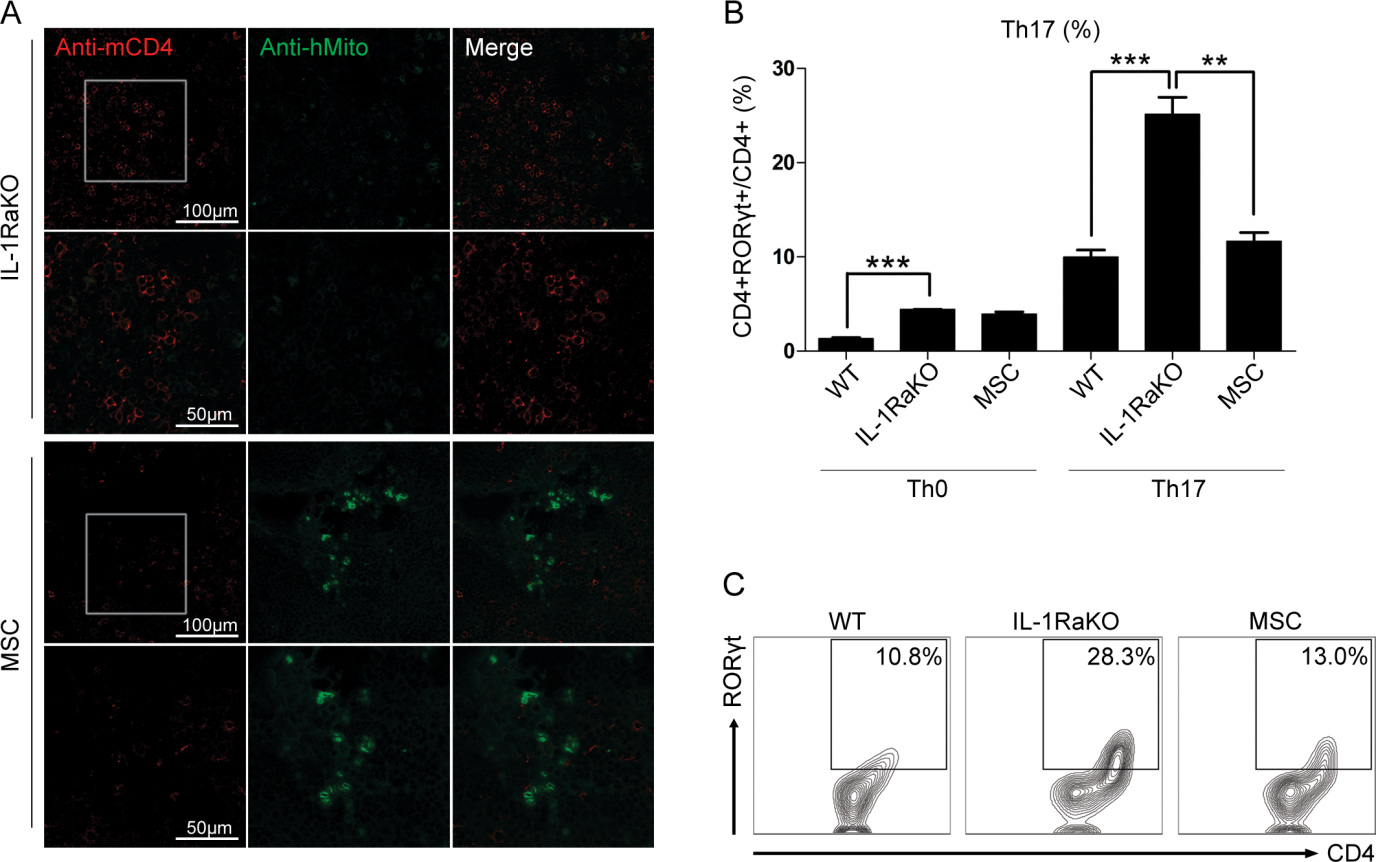
MSCs suppress Th17 differentiation of splenic CD4+ T cells. (A) Confocal fluorescence microscopy images (original magnification, 200×) of spleen sections (4 μm-thick) from IL-1Ra KO mice injected with MSCs immunostained with antibodies to human mitochondria (hMito) (green) and mouse CD4 (red). Scale bar: 100 μm (200×) and 50 μm (400×). (B) CD4+ T cells were isolated from the spleens of WT, IL-1RaKO, and IL-1RaKO mice injected with MSCs using anti-mouse CD4 microbeads. CD4+ T cells cultured under Th17-skewing conditions were compared with cells cultured under Th0 (non-skewing) conditions. After 3 days, CD4 and RORγt double-positive cells were analyzed by flow cytometry. (C) A representative dot plot from each group is shown. Error bars represent the mean ± SEM. *, P<0.05.

To localize human MSCs and mouse CD4+ T cells in the MSC group, spleen sections were stained with an anti-human mitochondria antibody and an anti-mouse CD4 antibody. In the MSC group, double-staining of MSCs (green) and mouse CD4+ T cells (red) was detected, demonstrating that human MSCs injected into IL-1RaKO mice can interact with mouse immune cells (Fig 3A). To determine the effects of MSCs on Th17 cell differentiation in IL-1RaKO mice, flow cytometry was used to analyze the Th17 differentiation ratio when splenic CD4+ T cells were cultured under Th17 polarizing conditions. There was no difference in the percentage of Th17 between IL-1RaKO and MSC group under Th0 conditions, but statistically significant differences were found under Th17 differentiation (Fig 3B). Fig 3C shows representative flow cytometry dot plots gated on the Th17 population. The differentiation of Th17 cells was significantly decreased in IL-1RaKO mice that received intraperitoneal injections of MSCs. Therefore, we have confirmed that the injected MSCs co-exist with splenic CD4+ T cells and affect Th17 polarization in IL-1RaKO mice.

### IL-1Ra expressed by MSCs can decrease Th17 differentiation

**Fig 4:**
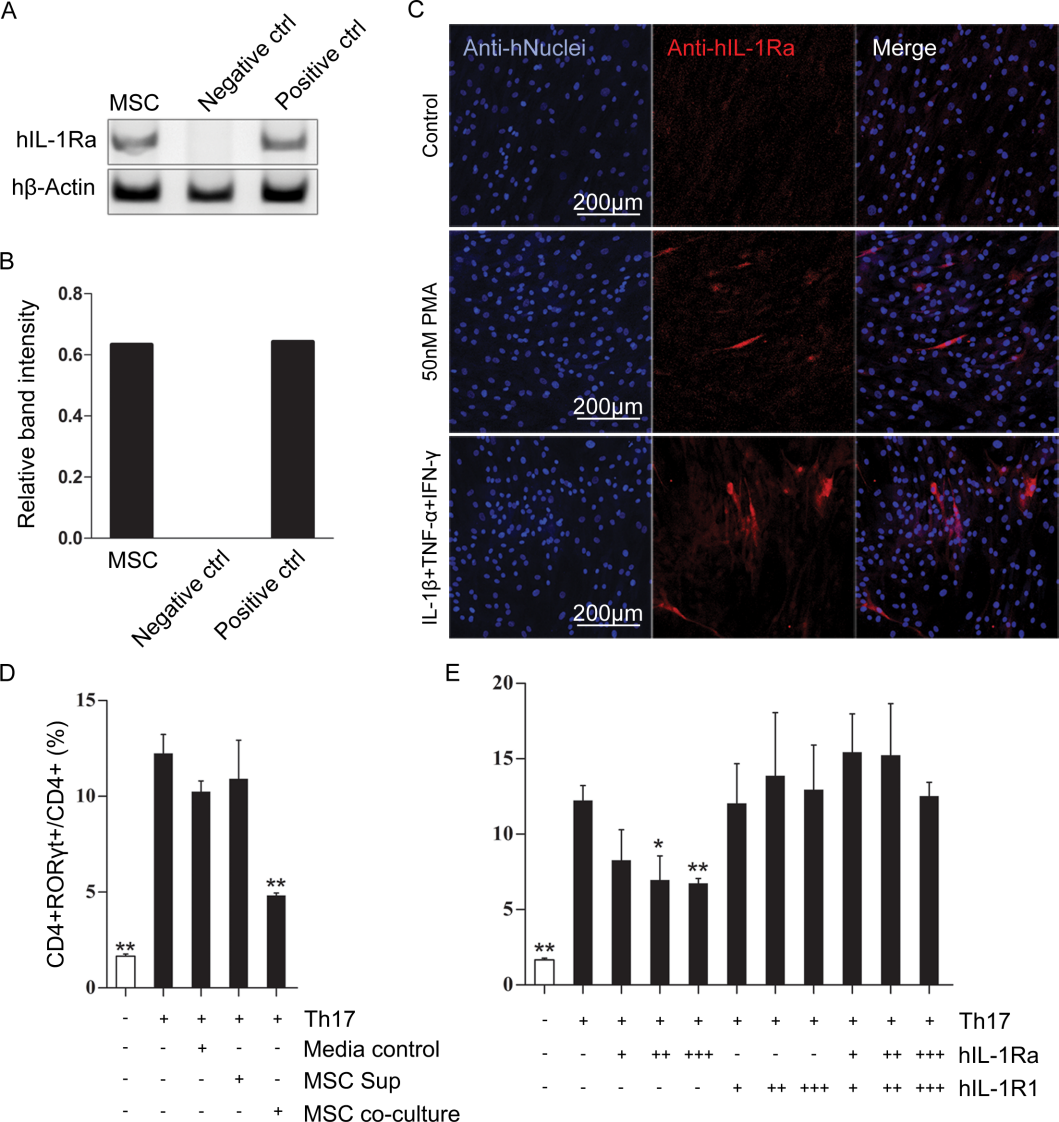
IL-1Ra expressed by MSCs decreased Th17 differentiation. (A) RT-PCR analysis of human IL-1Ra genes in MSCs, HEK293T and HeLa cells. (B) The densitometric quantification of the mRNA expression of human IL-1Ra. β-actin expression was used as control. (C) MSCs were stimulated with 50 nM PMA or 50 ng/ml IL-1β+TNF-α+IFN-γ for 48 h at 37°C. The expression of IL-1Ra was examined by IFA using a rabbit anti-human IL-1Ra antibody and an anti-rabbit Alexa Fluor594-conjugated secondary antibody. The human nuclei (hNuclei) were stained with DAPI. Original magnification: 100×, scale bar: 200 μm. (D-E) MSCs were co-cultured at 5×10^4^ cells per ml with 5×10^5^ cells CD4+ T cells. Human recombinant IL-1Ra (2, 5, and 10 ng/ml) or human IL-1 receptor type 1 (2, 5, and 10 ng/ml) was added to the wells under Th17-inducing conditions. After 3 days of co-culture, the differentiated Th17 cells were harvested and the proportion of Th17 cells was analyzed by flow cytometry as described above. IL-1Ra inhibited the differentiation of Th17 cells in a dose-dependent manner. Error bars represent the mean ± SEM. *, P<0.05. **, P<0.01.

HEK293T and HeLa cells were used as negative and positive control for the IL-1Ra gene, respectively. The mRNA level of human IL-1Ra was measured by RT-PCR in MSCs, HEK293T cells, and HeLa cells. Expression of IL-1Ra was detected in MSCs and HeLa cells, not HEK293T cells (Fig 4A). The mRNA levels of the IL-1Ra were quantified by densitometric analysis (Fig 4B). We conducted immunofluorescence assay (IFA) to confirm the expression for IL-1Ra at the protein level in MSCs. Expression of IL-1Ra protein did not be visually confirmed in normal MSCs. But, MSCs stimulated with pro-inflammatory cytokines expressed IL-1Ra according to the IFA (Fig 4C). To determine the effect of this human MSC IL-1Ra on differentiation of mouse Th17 cells, MSCs were co-cultured at a 1:10 ratio with CD4+ T cells isolated from spleens of IL-1RaKO mice under Th17 polarizing conditions (CD4-Th17 culture). The presence of MSCs in the CD4-Th17 culture significantly inhibited the expression of RORγt by flow cytometry (Fig 4D and 4E). In addition, a similar effect was observed when recombinant human IL-1Ra (rhIL-1Ra) was added to the CD4-Th17 culture. Under Th17 polarizing conditions, IL-1Ra dose-dependently decreased the generation of Th17 polarized cells. These data suggest that IL-1Ra expressed by MSCs inhibits Th17 cell polarization, which may contribute to the immunosuppressive function of MSCs.

## Discussion

Recently, MSCs have been studied in several animal models of autoimmune disease [25]. In this study, the immunomodulatory effects of MSCs in IL-1RaKO mice were studied. Interleukin-1 signaling is known to promote Th17 cell differentiation [31,32], and its natural anti-inflammatory antagonist, IL-1Ra, blocks the action of IL-1 functional ligands by competitive inhibition at the IL-1 receptor level [33]. Although MSCs are known to express IL-1Ra, the role of MSCs-derived IL-1Ra in the immunosuppressive mechanism of MSCs in mouse models of autoimmunity remained unclear. This study demonstrates that MSCs can express IL-1Ra in the inflammatory environment and can reduce inflammation by reducing the generation of Th17 cells in IL-1RaKO mice. This paper is the first to report that MSCs can inhibit inflammation in IL-1RaKO mice by expressing IL-1Ra.

In this study, we confirmed that the arthritic symptoms of IL-1RaKO mice were attenuated by the injection of human MSCs. The thickness in the hind paw was reduced, and histological analysis also showed reduction of cartilage degradation or hyperplasia (Fig 1). When we confirmed the population of Th17 cells and Treg cells ex vivo, the population of Th17 cells was reduced and that of Treg cells was increased in IL-1RaKO mice after MSC injection (Fig 2A). MSCs are known to secrete a multitude of anti-inflammatory cytokines and growth factors, including PGE2, TGF-β, and IL-10 [34]. While mouse IL-1Ra was not expressed, human IL-1Ra was expressed in joint of IL-1RaKO mice (Fig 2C). The level of human IL-1Ra expression was not that high, however, it proved the possibility of MSC migration in the mice joint. Recently, a subpopulation of MSCs that express IL-1Ra have been described [27]. In addition, MSCs secreted more of these factors in response to pro-inflammatory stimuli or hypoxia [35]. We evaluated cytokine production in the inflammatory microenvironment of IL-1RaKO mice that received MSCs. IL-1Ra expression was increased in MSCs exposed to IL-1β, TNF-α, and IFN-γ. In our previous study, MSCs were intraperitoneally injected into CIA mice, and observed in the liver, kidney, spleen, and even in the joint [30]. Several studies have demonstrated that intraperitoneal administration of human MSCs can attenuate inflammatory responses in mice [36,37]. The detection rate of MSCs was the highest in the spleen at 7 days post-injection [30]. An interaction between injected MSCs and splenic CD4 cells was also observed at 7 days post-injection. The presence of MSCs in spleen suggests that they may affect the immune system in mice.

With this observation, we stained the spleen of IL-1RaKO mice with human mitochondria antibody (Fig 3A). There was no expression of human protein in normal spleen of IL-1RaKO mice, however, GFP expression in spleen of MSC injected IL-1RaKO mice show that the MSCs were able to migrate to the spleen. The splenocytes of IL-1RaKO mice were more prone to Th17 differentiation compared to the MSC injected group (Fig 3B and 3C). Through this results we carefully suggest that the migrated MSCs may affect progenitor T cells, and suppress the differentiation of Th17 cells. In the study done by Luz-Crawford and colleagues, the interference capacity of MSCs with the functional activity of CD4+ T cells was confirmed [28]. The inhibition of proliferation of the activated cells and the cytokine production specific for Th17 was confirmed using activated CD4+ T cells cultured under in vitro conditions for Th17 lineage differentiation. Th17 cells induced the upregulation of PDL1, which played a critical role in inducing the immunosuppressive effect of MSCs. The authors also concluded that PDL1 supported the cell-to-cell contact dependence of MSC-mediated immune suppression on Th17 cells. Therefore, further study of the relation between MSC-T cell interaction and IL-1RaKO is required. Although we have not yet confirmed how the population of Treg increased, other factors besides IL-1Ra may be considered. It is known that GM-CSF enhances the immunosuppressive effect of Treg cells. GM-CSF is also a molecule known to be secreted in MSCs, similar to IL-1Ra. GM-CSF can be described as part of the immunosuppressive capacity of MSCs. Further research is needed on other factors such as GM-CSF [38–42].

IL-1RaKO exists in various forms [43]. To confirm which type of form might affect the T cell population, we co-cultured MSCs with CD4+ cells or treated MSC-cultured media to the CD4+ cells (Fig 4B). It was shown that the cultured media did not affect the Th17 cell population. However, co-culture of MSCs significantly reduced the differentiation of Th17 cells under in vitro conditions. The cell-to-cell contact of MSCs was previously studied by Ghannam et al [44]. To determine whether the effects were caused by cellular contact or secreted factors, the author co-cultured T cells and MSCs in a transwell system. The inhibition of IL-17 production by Th17 cells was abolished in transwell cell-culture systems, which indicates that direct contact is required for the inhibitory activity of MSCs.

Therapeutic agents that reduce the immune reaction in various autoimmune diseases are in the spotlight [45,46]. In this study, IL-1Ra, which can enhance the effect of MSCs in arthritis model, along with MSCs were selected and presented as a new therapeutic strategy concept. We confirmed the anti-inflammatory effects of MSCs in the IL-1RaKO mice model. IL-1Ra expressed by human bone marrow-derived MSCs could inhibit Th17 polarization and thereby decrease inflammation in IL-1RaKO mice. In conclusion, IL-1Ra may be one of the immune modulatory factors responsible for the anti-inflammatory actions of MSCs.
